# Supplementary material for: Causal Relationship between Aging and Anorexia Nervosa: A White-Matter-Microstructure-Mediated Mendelian Randomization Analysis
Source: Biomedicines. 2024 Aug 16;12(8):1874. doi: 10.3390/biomedicines12081874 (PMC11351342; doi:10.3390/biomedicines12081874)
Supplement: Supplementary file 1 [file biomedicines-12-01874-s001.zip › Supplementary Figure/Figure S4/Singe SNP plot of MR analysis for TL to ubm-b-1513.pdf]

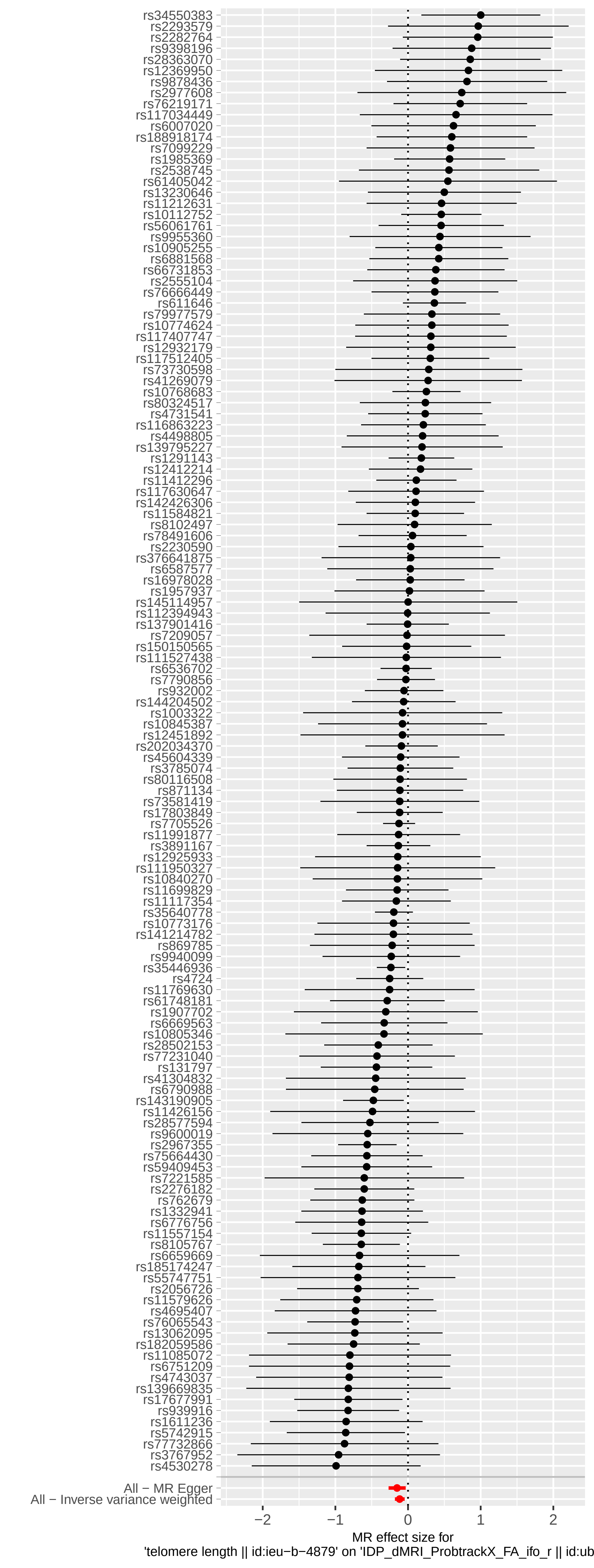

rs34550383
rs2293579
rs2282764
rs9398196
rs28363070
rs12369950
rs9878436
rs2977608
rs76219171
rs117034449
rs6007020
rs188918174
rs7099229
rs1985369
rs2538745
rs61405042
rs13230646
rs11212631
rs10112752
rs56061761
rs9955360
rs10905255
rs6881568
rs66731853
rs2555104
rs76666449
rs611646
rs79977579
rs10774624
rs117407747
rs12932179
rs117512405
rs73730598
rs41269079
rs10768683
rs80324517
rs4731541
rs116863223
rs4498805
rs139795227
rs1291143
rs12412214
rs11412296
rs117630647
rs142426306
rs11584821
rs8102497
rs78491606
rs2230590
rs376641875
rs6587577
rs16978028
rs1957937
rs145114957
rs112394943
rs137901416
rs7209057
rs150150565
rs111527438
rs6536702
rs7790856
rs932002
rs144204502
rs1003322
rs10845387
rs12451892
rs202034370
rs45604339
rs3785074
rs80116508
rs871134
rs73581419
rs17803849
rs7705526
rs11991877
rs3891167
rs12925933
rs111950327
rs10840270
rs11699829
rs11117354
rs35640778
rs10773176
rs141214782
rs869785
rs9940099
rs35446936
rs4724
rs11769630
rs61748181
rs1907702
rs6669563
rs10805346
rs28502153
rs77231040
rs131797
rs41304832
rs6790988
rs143190905
rs11426156
rs28577594
rs9600019
rs2967355
rs75664430
rs59409453
rs7221585
rs2276182
rs762679
rs1332941
rs6776756
rs11557154
rs8105767
rs6659669
rs185174247
rs55747751
rs2056726
rs11579626
rs4695407
rs76065543
rs13062095
rs182059586
rs11085072
rs6751209
rs4743037
rs139669835
rs17677991
rs939916
rs1611236
rs5742915
rs77732866
rs3767952
rs4530278
All − MR Egger
All − Inverse variance weighted
−2
−1
0
1
2
MR effect size for
'telomere length || id:ieu−b−4879' on 'IDP_dMRI_ProbtrackX_FA_ifo_r || id:ub
